# Detection, Molecular Identification and Transmission of the Intestinal Protozoa *Blastocystis* sp. in Guinea from a Large-Scale Epidemiological Study Conducted in the Conakry Area

**DOI:** 10.3390/microorganisms10020446

**Published:** 2022-02-15

**Authors:** Timothé Guilavogui, Nausicaa Gantois, Gaël Even, Jeremy Desramaut, Ellena Dautel, Constance Denoyelle, Fode Ibrahima Cissé, Salif Cherif Touré, Bakary Luther Kourouma, Manasi Sawant, Magali Chabé, Gabriela Certad, Eric Viscogliosi

**Affiliations:** 1 CNRS, Inserm, CHU Lille, Institut Pasteur de Lille, U1019—UMR 9017—CIIL—Centre d’Infection et d’Immunité de Lille, Université de Lille, F-59000 Lille, France; timotheguilavogui@gmail.com (T.G.); nausicaa.gantois@pasteur-lille.fr (N.G.); jeremy.desramaut@pasteur-lille.fr (J.D.); dautel.ellena@gmail.com (E.D.); constance.denoyelle@gmail.com (C.D.); manasi.sawant@pasteur-lille.fr (M.S.); magali.chabe@univ-lille.fr (M.C.); gabriela.certad@pasteur-lille.fr (G.C.); 2 Unité d’Appui à la Gestion et la Coordination des Programmes, Ministère de la Santé, Conakry BP 585, Guinea; 3 Gènes Diffusion, F-59501 Douai, France; g.even@genesdiffusion.com; 4 PEGASE-Biosciences (Plateforme d’Expertises Génomiques Appliquées aux Sciences Expérimentales), Institut Pasteur de Lille, F-59000 Lille, France; 5 Hôpital National Ignace Deen CHU de Conakry, Laboratoire de Parasitologie, 6ème Avenue—Kouléwardy, Kaloum, Conakry BP 1263, Guinea; epv59@hotmail.com (F.I.C.); touresalif17@gmail.com (S.C.T.); 6 Centre de Santé Anastasis, Quartier Nongo, Conakry BP 1032, Guinea; bakaryluther@gmail.com; 7 Délégation à la Recherche Clinique et à l’Innovation, Groupement des Hôpitaux de l’Institut Catholique de Lille, F-59000 Lille, France

**Keywords:** *Blastocystis* sp., intestinal protozoa, Africa, Guinea, molecular epidemiology, real-time quantitative PCR, SSU rDNA sequence, subtyping, transmission, zoonosis

## Abstract

*Blastocystis* sp. is a single-celled parasite estimated to colonize the digestive tract of 1 to 2 billion people worldwide. Although it represents the most frequent intestinal protozoa in human stools, it remains still under-investigated in countries with a high risk of infection due to poor sanitary and hygiene conditions, such as in Africa. Therefore, the present study was carried out to determine the prevalence and subtype (ST) distribution of *Blastocystis* sp. in the Guinean population. For this purpose, fecal samples were collected from 500 individuals presenting or not digestive disorders in two hospitals of Conakry. Search for the parasite in stools was performed by real-time PCR targeting the small subunit rDNA gene followed by sequencing of the PCR products for subtyping of the isolates. A total of 390 participants (78.0%) was positive for *Blastocystis* sp. Five STs were identified in the Guinean cohort (ST1, ST2, ST3, ST4 and ST14) with varying frequency, ST3 being predominant. Among them, ST4 was found in only two patients confirming its global rarity in Africa whereas infections by ST14 were likely the result of zoonotic transmission from bovid. No significant association was detected between *Blastocystis* sp. colonization or ST distribution and the symptomatic status of Guinean subjects or the presence of digestive symptoms. In contrast, drilling water consumption represented a significant risk factor for infection by *Blastocystis* sp. Predominance of ST3 coupled with its low intra-ST diversity strongly suggested large-scale human-to-human transmission of this ST within this cohort. In parallel, the highest intra-ST diversity of ST1 and ST2 was likely correlated with various potential sources of infection in addition to anthroponotic transmission. These findings highlighted the active circulation of the parasite in Guinea as reported in some low-income African countries and the necessity to implement prevention and control measures in order to limit the circulation of this parasite in this endemic geographical area.

## 1. Introduction

*Blastocystis* sp. is an anaerobic protist belonging to the highly diversified Stramenopile phylum that can colonize the intestine of humans and various groups of animals, including mammals, birds, reptiles or insects among others [1,2,3,4,5]. It has a very large distribution worldwide since it is estimated that over 1 billion people are colonized by this microorganism [6]. However, its prevalence is increased in underdeveloped countries that can easily be explained by its main mode of transmission, predominantly the fecal-oral route, via consumption of food or water contaminated with cystic resistance forms of the parasite [5,7]. This transmission is therefore directly correlated with the poor sanitary and hygienic conditions identified in low-income countries. Consequently, *Blastocystis* sp. frequencies reaching or exceeding 50% in African [8,9,10] or Asian [11,12] rural areas have been commonly reported, whereas the average prevalence of this enteric protozoan is around 15–25% in European industrialized countries, such as France [13], Spain [14] or Czech Republic [15].

The fact that the vast majority of individuals colonized by *Blastocystis* sp. does not present digestive disorders has ever questioned the potential pathogenicity of this microorganism [5,16,17,18]. However, studies using various in vitro systems and experimental rodent models [19] coupled with clinical case reports [20] clearly demonstrated the virulence of at least some isolates and their damaging effect on the intestinal epithelium of the host. In particular, these virulent isolates are able to induce apoptosis and degradation of tight junction proteins of targeted cells, resulting in increased intestinal permeability. Thus, taking also into account cumulative genomic data [21,22,23,24], current models of the interactions and pathways involved in *Blastocystis* sp. infection and pathogenicity have been proposed [19,25], potentially leading to non-specific intestinal symptoms such as diarrhea or abdominal pain and chronic hives. In parallel, recent findings showed that *Blastocystis* sp. colonization in humans was associated with changes in microbiota composition resulting in increased bacterial richness and, thus, in a healthy gut microbiome [26,27,28]. In contrast, *Blastocystis* sp. can decrease the abundance of some beneficial bacteria in a mouse model leading to a dysbiotic state [29].

Even if isolates of this parasite identified in different hosts mostly resemble each other morphologically [1,2,5], a very large genetic diversity has been observed within the genus *Blastocystis* based on the comparison of the small subunit (SSU) rDNA gene sequences. Briefly, 17 separate lineages so-called subtypes (STs) which may correspond to species were initially proposed among mammalian and avian isolates (ST1 to ST17) with different frequency in host species [30]. More recently, 15 additional STs (ST18 to ST32) were identified from birds, Bovidae and Cervidae [31,32,33,34,35,36]. However, four of these new STs (ST18-ST20 and ST22) are not currently considered as valid since they represent potential chimaeras arising during PCR amplification [37]. To complete this overview, various isolates identified in amphibians, reptiles, fish and insects and representative of the so-designed non-mammalian and avian STs (NMASTs) could also greatly expand the molecular heterogeneity observed in this genus [3,4,38]. Among all the STs identified, only 12 (ST1 to ST9, ST10, ST12 and ST14) were so far isolated from human stool with varying prevalence [10,39,40,41]. Indeed, ST1 to ST4 comprise more than 90% of all subtyped isolates around the world, with ST3 predominating in most countries [17,39]. On the other hand, the remaining STs are much more infrequent in the human population and with the exception of ST9, are likely the result of zoonotic transmission. Strong evidence is for instance supporting the zoonotic transmission of *Blastocystis* sp. ST5 and ST6 which are frequently harbored by hoofed animals and birds, respectively [42]. Indeed, isolates presenting identical sequences were shown to be shared by pigs and piggery workers for ST5 [43] and by chicken and slaughterhouse staff members for ST6 [44], in link with repeated and close contact with infected animals.

Despite the current status of *Blastocystis* sp. in terms of public health importance, its molecular epidemiology and circulation in Africa remains poorly investigated while populations of most African countries classified in majority as low-income countries are at high risk of parasitic infections mainly due to a lack of water sanitation. Accordingly, existing data on the prevalence of *Blastocystis* sp. in some of these countries, such as Senegal [10] and Nigeria [45] in West Africa, Cameroon [9] in Central Africa or Madagascar [46] in Southern Africa, revealed a high prevalence of over 60% in the corresponding populations. In other Western African countries such as Guinea, no molecular data on the prevalence and ST distribution of *Blastocystis* sp. were yet available. Therefore, the first epidemiological survey ever performed in this country was conducted on a large panel of 500 patients followed for different pathologies in two hospitals of Conakry and screened by real time Polymerase Chain Reaction (qPCR) for the detection of *Blastocystis* sp. The aim of the present study was thus to characterize the prevalence and STs of *Blastocystis* sp. in the Guinean population, then identify sources and modes of transmission of the parasite within this community.

## 2. Materials and Methods

### 2.1. Ethics Approval

The present on-site study in the Conakry area was approved by the National Ethics Committee on Health Research (CNERS) of Guinea (reference number 170/CNERS/20; date of approval: 24 December 2020). This study was conducted in accordance with the Code of Ethics of the World Medical Association (Declaration of Helsinki III) and with the International Ethical Guidelines for Biological Research Involving Human Subjects. After a clear explanation of the research objectives prior to enrolment, written informed consents were obtained from each adult or from the parents or guardians of minors participating in the study.

### 2.2. Questionnaire Survey

A standardized questionnaire was designed to collect information about each participant including gender, age, residence place, source of drinking water (drilling, tap or mineral water), contact with domestic animals and presence of digestive symptoms (i.e., diarrhea, abdominal pain, vomiting, bloating and constipation). A participant was considered symptomatic if at least one of the five selected digestive disorders described above was present. The subjects’ data were fully anonymized through the encryption of the identity of individuals.

### 2.3. Sampling Sites and Collection of Samples

**Figure 1:**
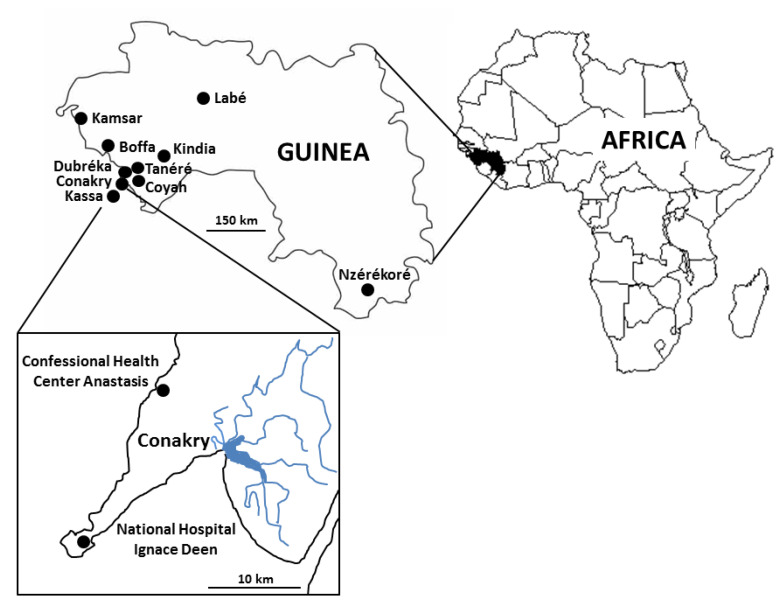
Map of Guinea showing the Conakry area where the study was conducted and the residence of the subjects enrolled in this study; the black box frames the two sites of participating hospitals: Confessional Health Center Anastasis and National Hospital Ignace Deen of Conakry.

This large-scale study was conducted in Conakry (geographical coordinates: latitude 9°32′16″ N, longitude 13°40′38″ W), the capital and largest city of Guinea, West Africa, with an estimated population of about 2,300,000 people (Figure 1). Conakry borders the Atlantic Ocean and is settled on Tombo Island, with the growing city spreading up the neighboring Kaloum Peninsula (36 km long and 0.2 to 6 km wide). The present study involved 2 hospitals of Conakry separated by ~15 km. The first and southernmost situated on the Tombo Island was the National Hospital Ignace Deen (NHID). The second one located in the north of the Conakry agglomeration on the Kaloum Peninsula was the Confessional Health Center Anastasis (CHCA). This survey was completed between January and March 2021. This period of the year corresponded to the dry season (January to April) under tropical monsoon climate. At this season, almost no precipitation falls in Conakry and the daily mean temperature reaches 26 to 27 °C.

Briefly, a cohort of 500 patients followed up at the two hospitals for different pathologies, with/without gastrointestinal symptoms, were enrolled (Table 1). A total of 250 stool samples (1 sample per patient) were collected at each participating center during routine clinical procedures. The majority of the subjects participating in this study (81.0%, 405/500) lived in Conakry while the other participants came from towns in the suburbs of Conakry, such as Coyah (2 subjects), Tanéré (1), Kassa (1) and Dubréka (12) or from towns farther away, such as Kindia (74), Boffa (1), Kamsar (2), Labé (1) and Nzérékoré (1). The significant number of subjects followed in CHCA and living in Kindia could easily be explained by the proximity of this hospital to this city.

For each participant, around 2 g of fresh stools was gathered and then homogenized by shaking in 2 mL of 2.5% potassium dichromate (*w*/*v* in water) (Sigma Life Sciences, Saint-Louis, MO, USA) in a sterile tube. All samples were stored at 4 °C and then transported to the Institut Pasteur of Lille (France) for DNA extraction and molecular screening and subtyping of *Blastocystis* sp.

### 2.4. DNA Extraction and Molecular Subtyping of Blastocystis sp. Isolates

For the detection of *Blastocystis* sp., 1 mL of stool diluted in potassium dichromate was centrifuged, then washed 3 times for 10 min at 3000 g with water. The resulting pellet was diluted in 500 µL of water, then used for total genomic DNA extraction using the NucleoSpin 96 Soil kit or NucleoSpin Soil, Mini kit for DNA from Soil (Macherey-Nagel GmbH & Co KG, Düren, Germany) according to the manufacturer’s recommendation. DNA was eluted in 100 μL of elution buffer provided in the DNA extraction kits and stored at −20 °C until further processing. For each sample tested, 2 μL of the obtained DNA templates was examined for the presence of the parasite by qPCR using the *Blastocystis*-specific primers BL18SPPF1 (5′-AGTAGTCATACGCTCGTCTCAAA-3′) and BL18SR2PP (5′-TCTTCGTTACCCGTTACTGC-3′) as described [47]. The amplified fragment of approximately 300 bp length of the SSU rDNA gene has been shown to contain sufficient sequence information for accurate subtyping of *Blastocystis* sp. isolates [10,13,38,41,44]. Both positive (DNA obtained from *Blastocystis* sp. ST8 axenic culture) and negative (DNA matrix replaced by water) qPCR controls were included. The positive qPCR products were purified and directly sequenced in both directions (Genoscreen, Lille, France; SANGER technology platform, 3730XL DNA Analyzer). In case of sequence chromatograms with double traces suggesting infections by at least two different *Blastocystis* STs, the corresponding STs were not determined and these positive samples were considered as mixed infections. The SSU rDNA sequences obtained in this study were deposited in GenBank under accession numbers OM038693 to OM038983. The STs of the new sequences were identified by determining the exact match or closest similarity against all *Blastocystis* sp. homologous sequences of known STs available from the National Centre for Biotechnology Information (NCBI) using the nucleotide Basic Local Alignment Search Tool (BLAST) program. Additionally, the sequences of *Blastocystis* sp. isolates belonging to ST1, ST2 or ST3 were aligned with each other using the BioEdit v7.0.1 package (date of release 10 June 2019; http://www.mbio.ncsu.edu/BioEdit/bioedit.html, accessed on 12 January 2022) to determine intra-ST diversity and identify so-called genotypes referring to genetically distinct strains within the same ST [10,41].

### 2.5. Statistical Analysis

For the statistical analysis, Fisher’s exact test was used to test the relationship between different categorical variables. Multilevel logistic mixed regression models were created to calculate odds ratios (OR) and 95% confidence interval (CI) considering *Blastocystis* sp. colonization, STs and genotypes as the main outcomes. The general significance level was set at a *p*-value below 0.05. All analyses were performed using packages stats and oddsratio 2.0.1 from the R statistical computing program (Version 4.1.1, date of release 8 October 2021; R Development Core Team, http://www.R—project.org, accessed on 12 January 2022).

## 3. Results

### 3.1. Analysis of the Cohort of Guinean Subjects, Prevalence and Risk Factors Associated to Blastocystis sp. Infection

Stool samples were obtained randomly from 250 male and 250 female patients (sex ratio M/F of 1.0) and the age of participants ranged from 1 to 83 years (mean age of 26 ± 17 years). Among this cohort, 433 subjects (86.6%) were considered symptomatic since they presented at least one of the 5 selected digestive symptoms. Abdominal pain was by far the most frequent digestive disorder among symptomatic patients (380/433, 87.8%), followed by constipation (133/433, 30.7%), diarrhea (58/433, 13.4%) and vomiting (18/433, 4.2%). No patient complained of bloating. The remaining 67 individuals (13.4%) were identified as asymptomatic at the time of the study.

Of the 500 stool samples tested by qPCR, the overall prevalence of *Blastocystis* sp. was shown to be 78.0% (390/500) (Table 2). By analyzing the data separately for each hospital, the average prevalence observed in the CHEA (77.6%) was not significantly different to that determined in the NHID (78.4%) (Fisher exact test, *p* = 0.9141). Considering only the 2 more frequent places of residence of the patients that were Conakry (405 patients and 77.5% of positive participants) and Kindia (74 subjects and 79.7% of positive subjects), no significant risk for parasite infection was found associated to these towns of residence (OR: 0.913, CI:0.504-1.585, *p* =0.755 and OR:1.145, CI:0.635-2.176, *p* = 0.665, respectively).

Within the global cohort, the difference of prevalence observed between males (196/250, 78.4%) and females (194/250, 77.6%) was not significant (Fisher exact test, *p* = 0.914). In parallel, the age subgroup analysis showed that the prevalence of *Blastocystis* sp. was 80.6% (162/201) in the group aged 0–18 years, 83.0% (93/112) among subjects aged 19–30 years, and 72.2% (135/187) in patients aged over 30 years. Multilevel logistic mixed regression model revealed that patients over 30 years had significantly less risk of being colonized by *Blastocystis* sp. (OR: 0.59, CI: 0.385–0.908, *p* = 0.016) compared to the 0–18 and 19–30 years subgroups. The analysis also showed that contact with animals was not a risk factor associated with the presence of *Blastocystis* sp. (Fisher exact test, *p* = 0.285). In contrast, drilling water consumption represented a significant risk factor for infection since drilling water consumers had 2 times higher risk of *Blastocystis* sp. infection than non-consumers (82.0% of positive subjects versus 71.2%; OR: 1.838, CI: 1.196–2.825, *p* = 0.005). Additionally, consumption of tap water (Fisher exact test, *p* = 0.746) or mineral water (Fisher exact test, *p* = 0.914) were not shown to be protective factors against *Blastocystis* sp. infection among examined patients. Regarding parasite infection and digestive symptoms, the prevalence of *Blastocystis* sp. was not reported to be significantly higher in symptomatic patients than in asymptomatic carriers (78.1% versus 77.6%; Fisher exact test, *p* = 1.000). Similarly, the parasite was not identified more frequently in subjects presenting one of the digestive symptoms reported in more than 20 individuals, whether it was abdominal pain (77.9 versus 78.3% in asymptomatic participants; Fisher exact test, *p* = 0.919), diarrhea (75.9 versus 78.3%; Fisher exact test, *p* = 0.736), or constipation (76.7 versus 78.5%; Fisher exact test, *p* = 0.714).

### 3.2. Distribution of Blastocystis sp. STs

Among the 390 positive samples, 99 of them (25.4%) were shown to correspond to mixed infections through the identification of double traces on the respective sequence chromatograms (Table 2). This proportion of mixed infections was very similar between the two hospitals (24.7% for the CHCA versus 26.0% for the NHID). The remaining 291 positive samples represented infections by a single ST. Among this latter group of patients, ST3 was predominant (117/291, 40.2%) followed by ST1 (96/291, 33.0%), ST2 (66/291, 22.7%) and ST14 (10/291, 3.4%). ST4 was identified in only two participants (2/291, 0.7%). In total, 5 different STs were detected in this Guinean cohort and found in each of the hospitals with varying prevalence.

Indeed, the distribution of predominant *Blastocystis* sp. STs (ST1 to ST3) varied widely between the two hospitals since the risk of ST1 infection was significantly lower in the NHID than in the CHCA (17.3% of the single infections versus 42.5%; OR: 0.447, CI: 0.275–0.716, *p* = 0.001) while the risk of ST3 infection was almost two times higher in the NHID (46.9 versus 33.6%; OR: 1.572, CI: 1.017–2.443, *p* = 0.042). Moreover, although ST2 was also more frequently identified in the NHID than in the CHCA (26.9 versus 18.5%), there was not more risk of being infected in one hospital compared to the other one (OR: 1.536, CI:0.902–2.650, *p* = 0.117). Additionally, considering the two most frequent cities of residence, subjects from Kindia were significantly more at risk of being carriers of *Blastocystis* sp. ST1 than those from Conakry (39.0% versus 22.0%; OR: 2.269, CI: 1.249–4.064, *p* = 0.006) (Table 3). Moreover, there were not significant differences between the residents of Kindia and Conakry concerning the colonization by ST2 (19.0 versus 17.2%; OR: 1.103, CI: 0.516–2.195, *p* = 0.788) or ST3 (22.4 versus 31.8%; OR: 0.605, CI: 0.302–1.140, *p* = 0.135).

The distribution of STs was not significantly associated with the sex of the participants (Fisher exact test, *p* = 0.737) in contrast with age. Indeed, analysis of the subjects revealed that ST1 was more frequently reported in the group aged 0-18 years (OR: 2.092, CI: 1.314–3.348, *p* = 0.002) and less commonly found in patients aged over 30 years (OR: 0.476, CI: 0.275–0.798, *p* = 0.006). On the other hand, no significant difference of prevalence of either ST2 (Fisher exact test, *p* = 0.119) or ST3 (Fisher exact test, *p* = 0.301) was demonstrated between age groups. Regarding drinking water, our data showed that mineral water consumers were significantly less colonized by ST1 (OR: 0.528; CI: 0.329–0.84, *p* = 0.007) as were those drinking tap water (OR: 0.397; CI: 0.245–0.634, *p* = 0.0001). In contrast, the risk of ST2 infection increased twofold in the group of patients drinking drill water (OR: 2.335; CI: 1.257–4.633, *p* = 0.01). No significant association was detected between ST1 (OR: 0.628, CI: 0.338–1.204, *p* = 0.149), ST2 (OR: 1.139, CI: 0.534–2.725, *p* = 0.751) or ST3 (OR: 1.066, CI: 0.570–2.082, *p* = 0.845) and the symptomatic status of the Guinean subjects as well as between each of these STs and digestive symptoms, including abdominal pain (Fisher exact test, *p* = 0.926), constipation (Fisher exact test, *p* = 0.766), and diarrhea (Fisher exact test, *p* = 0.760).

### 3.3. Identification of Blastocystis sp. Genotypes and Analysis of Intra-ST Diversity

**Figure 2:**
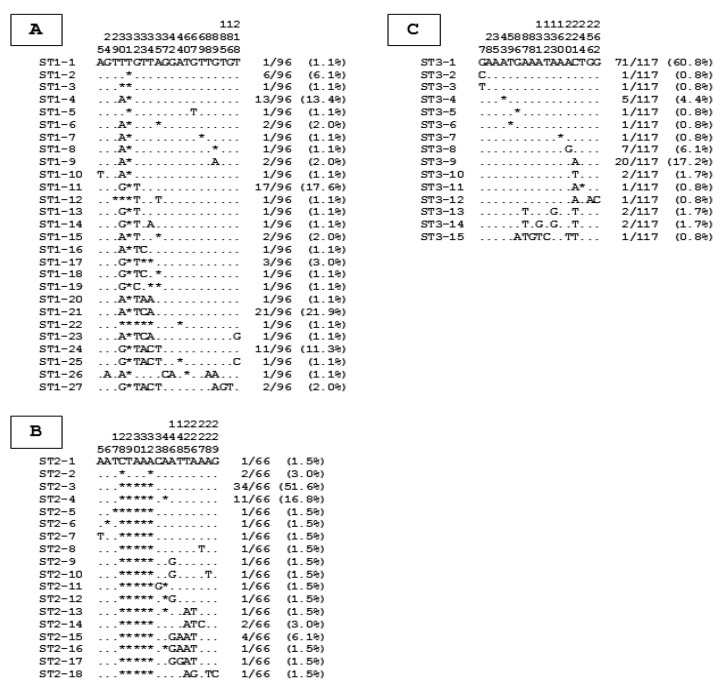
Alignment of partial SSU rDNA gene sequences from *Blastocystis* sp. ST1 (**A**), ST2 (**B**), and ST3 (**C**) isolates. Only the variable positions identified in the compared domain of the SSU rDNA gene for these three STs highlighted as largely predominant in the present survey are shown in this alignment. The positions of variable positions with respect to the reference sequences (genotypes ST1-1, ST2-1 and ST3-1) are indicated above the alignment (vertical numbering). Nucleotides identical to those of the reference sequences are represented by dashes and gaps are represented by asterisks. All the genotypes identified for each ST are indicated on the left of the alignment. On the right of the alignment are reported the total number and percentage of isolates identified in our study for each genotype.

All the partial SSU rDNA gene sequences belonging to each of the three main STs, ST1 to ST3, were aligned with each other to identify genotypes and evaluate intra ST-diversity. Regarding ST1, the 96 sequences obtained in the present study exhibited 95.5 to 100% identity between them. Through the alignment of these latter sequences, a total of 20 positions showing at least one nucleotide difference within at least one of the compared sequences, was highlighted. In total, 27 genotypes so-called ST1-1 to ST1-27 (Figure 2A) were identified. The majority of these ST1 genotypes (21 of 27) was represented by 1 or 2 isolates. However, 4 of these genotypes (ST1-4, ST1-11, ST1-21 and ST1-24) accounted for nearly 65% of the ST1 isolates. About ST2, 17 variable positions were identified from the sequence alignment of the 66 isolates exhibiting 96.5 to 100% identity, leading to the characterization of 18 genotypes (ST2-1 to ST2-18) (Figure 2B). As for ST1, most of the ST2 genotypes included 1 or 2 isolates and few other genotypes (ST2-3 and ST2-4) were predominant (more than 65% of the ST2 isolates). In the case of the 117 ST3 isolates, their corresponding SSU rDNA gene sequences showed 98.6 to 100% identity between them and 17 variable positions were reported from the alignment, leading to the identification of 15 genotypes (ST3-1 to ST3-15) (Figure 2C). Two of them (ST3-1 and ST3-9) were overabundant by including more than 75% of the ST3 isolates, the others genotypes being largely less represented. By calculating the ratio between the number of isolates and the number of genotypes for each ST, we revealed an average of 3.6 isolates per ST1 genotype (96/27), 3.7 isolates per ST2 genotype (66/18) and 7.8 isolates per ST3 genotype (117/15).

Between the 2 compared hospitals, the number of genotypes identified was roughly similar since a total of 34 genotypes (10 ST1, 13 ST2 and 11 ST3 genotypes) was reported in the NHID versus 39 (22 ST1, 8 ST2 and 9 ST3 genotypes) in the CHEA. Moreover, as numerous genotypes included 1 or 2 isolates, only 13 genotypes (ST1-2, ST1-4, ST1-11, ST1-21, ST1-24, ST2-3, ST2-4, ST2-15, ST3-1, ST3-4, ST3-8, ST3-9 and ST3-10), most of them overrepresented in the present survey were shared in both hospitals. Despite the large number of genotypes identified, only one of them, ST1-4 showed relevant variations in prevalence according to different factors since it was less frequently reported in patients followed at the NHID (OR: 0.158, CI: 0.024–0.609, *p* = 0.019), in symptomatic subjects (OR: 0.239, CI: 0.073–0.844, *p* = 0.019), in participants presenting abdominal pain (OR: 0.322, CI: 0.101–1.05, *p* = 0.052) or drinking tap water (OR: 0.158, CI: 0.024–0.609, *p* = 0.018). In contrast, the risk of ST1-4 infection was higher among people living in Kindia than in Conakry (OR: 10.4, CI: 3.210–36.900, *p* = 0.0001) and in subjects with animal contact (OR: 6.276, CI: 1.953–20.818, *p* = 0.002). The risk of infection with a second genotype, ST3-1 was also significantly higher in adults over 30 years than in the other age groups (OR: 2.375, CI: 1.294–4.392, *p* = 0.005).

Concerning the other STs identified in our study with lower frequencies such as ST4, the sequences of the 2 isolates exhibited 99.3% identity (2 variable positions). About the 10 ST14 isolates, the corresponding sequences showed 99.6 to 100% identity between them (only 1 variable position).

## 4. Discussion

To our knowledge, the present survey performed in Guinea represents the second largest epidemiological study conducted to date in Africa on *Blastocystis* sp. in terms of the number of subtyped isolates (291 in total herein) after the one recently conducted in Senegal [10], thus providing a significant new contribution to our understanding of the prevalence and circulation of this intestinal protozoan in African developing countries. Through the screening by qPCR of fecal samples from 500 individuals followed in two hospitals of Conakry, a prevalence of *Blastocystis* sp. of as much as 78% has been reported, highlighting the active circulation of this parasite in this geographical area. The burden of *Blastocystis* sp. in the Guinean population was all the more significantly relevant if we consider that the sampling was carried out during the dry season (winter) and not over the rainy season (summer), which is known for facilitating the transmission of this waterborne parasite [48]. In addition, for practical reasons only a single stool sample per individual was screened, instead of the ideal three consecutive samples needed for reliable detection of this parasite with irregular shedding [49].

All epidemiological data collected in this survey were compared with those obtained only by molecular assays in other countries and regions of Africa (Table 4). Indeed, it is known that findings recorded from non-molecular diagnostic methods greatly underestimate the prevalence of *Blastocystis* sp. [47,50]. West Africa is the African region gathering most prevalence data, as several studies have been conducted in Côte d’Ivoire with a prevalence ranging from 58.2% to 87% [48,51,52], in Nigeria with a prevalence of 49% [39], 84% [45] and 55% [53], in Liberia with a prevalence of around 70% [39], in Senegal with a prevalence of 80.4% in the Saint-Louis area [39] and 100% in Podor district [8] and in Mali with a prevalence of 49.7% [54].

Regarding other African regions, the number of studies is extremely limited with very few countries investigated. In Central Africa, the prevalence of *Blastocystis* sp. remains quite low in Angola since it is about 25% [55] but largely higher in Cameroon by exceeding 75% in two separate surveys [9,28]. In East Africa, four studies were conducted in Tanzania revealing prevalence comprised between 53% and 81.8% [9,24,50,53], depending of the geographical area. One of these studies also included a Sudanese cohort with a prevalence of *Blastocystis* sp. reaching 47% [53]. In Southern Africa, three studies were performed in Madagascar, Mozambique and Malawi showing a parasite prevalence of 64.5% [46], 14% [56] and 69.6% [57], respectively. In North Africa, no less than 15 molecular studies have been referenced in the literature (Table 4), but only one of them, conducted in Libya, provided a parasite frequency with a value of about 28% [39]. Globally, and even though substantial differences in prevalence rates may occur between countries, such as between Senegal and Mali in West Africa, or even in the same country, such as in Nigeria or Tanzania, that could be attributed to different factors including climatic factors, environmental hygiene and socio-economic conditions among others, the majority of the African surveys emphasized a parasite prevalence of around or well over 50%, in correlation with the frequency observed in Guinea. In comparison, the prevalence of *Blastocystis* sp. is significantly much lower in high-income nations including, for instance, Western [15] and Eastern [13,14,58] European countries, exhibiting an average prevalence ranging between 15 and 25%. Therefore, the significant prevalence values of *Blastocystis* sp. reported in African developing countries are undoubtedly associated with fecal peril in link with precarious sanitary and hygiene conditions and poor quality of drinking water.

These findings highlight the importance of screening the presence of this parasite in other not yet documented countries, to better estimate its burden among the global African population. As examples among others, *Blastocystis* sp. would be by far the most common intestinal protist in Algeria (prevalence of 57.3 and 43.8% in two separate studies) [74,75], Morocco (64.0%) [76] and Zambia (53.8%) [77], based solely on non-molecular methods of detection (microscopic analysis of stools supplemented or not by concentration and staining).

Interestingly, and even though the composition of the cohorts in each of the two Guinean hospitals was slightly different, particularly in terms of the place of residence of the participants as almost all individuals living in Kindia were followed in the CHCA (70/72), the prevalence of *Blastocystis* sp. was very similar between these two health centers, suggesting an intense circulation of the parasite in Conakry as well as in the close periphery of the Guinean capital. Among the overall Guinean cohort, gender and contact with animals were not identified as risk factors for infection by *Blastocystis* sp. In contrast, the prevalence of the parasite was higher in the age classes 0–18 and 19–30 years (more than 80% in both classes) in comparison to individuals over 30 years (~70%). Conflicting results regarding the frequency of *Blastocystis* sp. according to age were reported in the literature as for instance in African countries. No significant difference in prevalence among age classes was thus observed in Côte d’Ivoire [48], while the frequency of the parasite increased significantly with age in Tanzania [50] as in Nigeria [45] but only within children aged 2–14 years in this latter survey. All of these data were thus not consistent with the results of the present study revealing a higher prevalence in younger participants. This distribution in the Guinean population with a higher prevalence in the group 0–18 years old could likely be explained by inappropriate hygiene practices of younger individuals, such as lack of hand washing before meals or after toilet use, fingernail cleanliness, open filed defecation or playing in contaminated water leading to possible frequent reinfections through different sources. Moreover, promiscuity in schools represents an additional factor facilitating the transmission of the parasite within younger participants. Regarding the lower frequency of *Blastocystis* sp. in the group over 30 years, it could be due to the better understanding and application of personal hygiene measures by adults.

The findings of this study also suggest that the consumption of untreated drill water is significantly associated with increased odds of *Blastocystis* infection. This is logical considering that water could be a major source of infection by this waterborne parasite as described in other African countries [45]. Unexpectedly, the consumption of mineral water was not a protective factor against *Blastocystis* sp. infection, which contradicted observations recorded, for instance, in Nigeria, where the prevalence of the parasite was significantly lower among consumers of sachet water than among those drinking water collected from wells [45]. This could be attributed to the fact that mineral water is likely not the only source of consumption of Guinean participants, especially outside the home. Tap water supply was also not protective against parasite infection in the Guinean cohort probably in link with inadequate treatment of water for human consumption. However, when considering risk factors for each ST, mineral water and tap water consumers were significantly protected of being colonized by ST1.

Interestingly, *Blastocystis* sp. was not more prevalent in the symptomatic group as also in patients presenting digestive symptoms, including diarrhea, abdominal pain or constipation. The present survey together with others previously conducted in various African countries, including Madagascar [46], Tanzania [50] and Côte d’Ivoire [48], did not reveal any clear association between *Blastocystis* sp. infection and gastrointestinal disorders, raising again the question of the real pathogenic potential of this parasite, at least for a majority of the isolates, as previously discussed in details [18]. However, such a correlation can scarcely be demonstrated in surveys conducted in African countries whose populations are often co-colonized with other intestinal protozoa (*Giardia*, *Cryptosporidium* and *Entamoeba*, among others) [46,50,51,52] that can cause the same digestive symptoms as those associated with *Blastocystis* sp. infection.

As stated above, 99 of the 390 positive samples screened herein corresponded to mixed infections. This proportion (~25%) was similar to that reported in a neighboring country such as Senegal (23%) [10]. The remaining Guinean participants presenting single infections were colonized by ST3 (40.2% of the subtyped isolates), ST1 (33.0%), ST2 (22.7%), ST14 (3.4%) and ST4 (0.7%). In West Africa, the three main STs globally exhibit rather similar frequencies, with a slight predominance of ST2 (Table 4). The situation in terms of prevalence of the three main STs is more or less the same in East Africa, with a moderate higher prevalence of ST1. In Southern Africa, the prevalence of ST1 and ST3 are almost identical while ST2 is much less frequent. In contrast, ST3 is largely predominant in North Africa and to a lesser extent in Central Africa. Due to the still limited number of surveys and subtyped isolates in most of the African regions, these observations only correspond to tendencies most often associated with non-significant differences in ST frequencies, the only possible exception being the Maghreb where ST3 prevalence is far ahead of those of the other main STs. Indeed, extensive variations in the distribution of STs were documented within a same country, as in Nigeria [39,45] or Senegal [8,10], which are certainly related to different sources of transmission of the parasite. On the other hand, this distribution was similar in two Cameroonian cohorts [9,28] even though the respective studies were conducted in different geographical regions and among populations presenting various subsistence modes. In the present study, a significant variation in the distribution of STs was also observed between the two hospitals of Conakry highlighted by a higher prevalence of ST3 and lower frequency of ST1 in the NHID. This variation could probably be explained in part by the difference in the composition of the two hospital cohorts regarding the participants’ place of residence since the large majority of subjects living in Kindia were followed in the CHCA and significantly more colonized with ST1 than the inhabitants of Conakry.

Knowing which *Blastocystis* sp. STs circulate in Guinea may provide evidence of the pathways involved in transmission. According to our present data, ST1, ST2, and ST3 isolates represent the large majority of isolates identified in Guinea (95.9%) as more globally in Africa (2400/2527, 95.0%) (Table 4). These three STs can also be found in various groups of animals with varying frequency [42]. However, they predominantly colonize humans, irrespectively of the geographical origin of the population as reported in Europe, America or Asia [16,17,39], reflecting thus a large-scale human-to-human transmission of these STs. This was also strongly suggested herein in the Guinean cohort as in previous surveys conducted in different African countries [10]. In contrast, ST4 was identified at a much lower rate in the Guinean population since only two ST4 isolates were reported (0.7%). Interestingly, only 45 isolates of ST4 have been recorded in Africa out of the 2527 isolates subtyped so far on this continent (Table 3), i.e., barely 1.8% of the total number of African isolates. In comparison, this ST is frequently found, particularly in Europe where it can reach a prevalence of ~20% [13,14,39,47]. This geographically restricted pattern of ST4 thus tends to confirm the hypothesis of the recent emergence of ST4 in the European population [16], probably through zoonotic transmission from rodents, in which ST4 is the most common ST [42]. In this respect, nearly half of the African ST4 isolates (20/45) have been identified in the Maghreb and thus in geographical proximity to the European continent. Another interesting point of our survey was the report of ST14 isolates in a significant proportion (3.4%). Indeed, together with ST10, ST14 corresponds to the most widely distributed ST in cattle as to a lesser extent in small ruminants [42], indicating that both STs could be considered as adapted to bovid [42,78,79]. Therefore, bovid living in close proximity to dwellings likely represent zoonotic sources of ST14 colonization in the Guinean population. However, 9 of the 10 participants colonized with ST14 reported any direct contact with animals suggesting that these subjects were sporadically colonized by ST14 through the punctual consumption of water or food contaminated by bovine feces. The remaining participant declared having contact with chickens and more interestingly with sheep, which may be at the origin of this ST14 contamination. To our knowledge, this is the second report about ST14 identification in the human population, the first one concerned the infection of two school children living in rural areas in Senegal [10].

No significant difference in the distribution of *Blastocystis* sp. STs between the sexes was reported herein as shown, for instance, in Tanzania [50]. On the other hand, ST1 was more common in the youngest age group of Guinean participants but not significantly. Furthermore, ST2 and to a lesser extent ST1 were the most common STs in subjects drinking untreated drill water, highlighting the likely waterborne transmission of a large majority of ST1 and ST2 isolates in the Conakry area. Interestingly, the transmission of these same two STs was also suggested to be mainly associated with different environmental sources in a recent study conducted in Senegal [10]. In parallel, the epidemiological data collected in Guinea failed to provide any evidence for ST association with symptomatic status or specific digestive symptoms of the subjects as also described in Côte d’Ivoire [48]. For different reasons, this correlation between ST and pathogenesis of the parasite remains globally contradictory according to numerous studies [5].

To complete our data and taking advantage of the large number of isolates subtyped in the present survey, the intra-ST variation observed within the 3 major STs identified in the Guinean population was analyzed. This analysis succeeded those recently performed in Senegal [10] and among Syrian refugees leaving in North Lebanon [41] using the same molecular marker and comparing the sequence of the same amplified domain. By first determining the genotypes for each of these 3 STs through the identification of the variable positions between sequences of isolates belonging to the same ST and, then, by establishing the ratio of the number of isolates per genotype, it was shown that ST3 exhibited the lowest intra-ST diversity followed by ST2 and ST1 in the Senegalese and Syrian cohorts [10,41]. Strikingly, a similar pattern of intra-ST diversity was highlighted for ST1 to ST3 among Guinean isolates. Moreover, the higher intra-ST variability of ST1 and ST2 compared with ST3 was also confirmed through the comparative analysis of draft genomes from various STs of *Blastocystis* sp. [24].

As stated above, a total of 60 ST1 to ST3 genotypes were identified in the Guinean population for a total of 279 isolates belonging to these 3 STs, i.e., an overall average of 4.65 isolates per genotype. In a Senegalese cohort, this number of genotypes was significantly lower (43 different genotypes) for a total of 446 isolates from ST1 to ST3 leading to a higher average ratio of 10.4 isolates per genotype [10]. This average ratio was further increased in a Syrian refugee cohort to 13.6 isolates per genotype due to the low number of identified genotypes (only 12 in total) [41]. The high number of isolates per genotype coupled to the limited number of genotypes in the Syrian cohort could be explained by sustained human-to-human transmission within this group in link with promiscuity in refugee camps and limited circulation of this population outside the informal tented settlements. In case of the Senegalese survey, the increasing number of genotypes compared to the Syrian cohort together with the lower number of isolates per genotype suggested the existence of multiple potential sources of infection coupled with a large anthroponotic transmission. These two parameters, meaning the number of genotypes and the number of isolates per genotype, therefore represented interesting transmission markers within the populations studied. Thus, the Guinean population, with its even higher number of genotypes and lower ratio of isolates per genotype, showed active human-to-human transmission, but also, and more importantly, an even wider range of potential sources of infection than those described in Senegal. While human-to-human transmission is mainly related to ST3 since it is not frequently found in animals [42], ST1 and ST2 which represented the largest number of genotypes often including 1 or 2 isolates were likely transmitted in large part from various environmental and possibly animal reservoirs in Guinea. In this respect, ST1 was identified as the most widespread ST from various water sources in Asiatic countries [80]. Further studies will thus allow analyzing samples from these potential animal and environmental sources for the presence of the parasite.

## 5. Conclusions

This study provides the first epidemiological data on the prevalence and distribution of *Blastocystis* sp. STs in the Guinean population and further clarifies the current burden and circulation of this parasite in the African continent. The picture is of concern and the prevalence reaching 80% in Guinea simply reinforces this statement as already pointed out in other African countries. Our findings suggest that age and drill water consumption are risk factors associated with *Blastocystis* sp. infection. Even if the consumption of either mineral or tap water were not protective factors against *Blastocystis* sp., these factors were indeed associated with reduced odds of ST1 infection.

The epidemiological situation in Guinea is probably correlated to the fecal peril in link with precarious hygiene and sanitary conditions encountered among the populations of these low-income countries. Moreover, the poor quality of drinking water mainly due to deficient sanitation results in the exposure of the population to multiple contaminated environmental sources, facilitating the circulation of this waterborne parasite. As in all African countries and regions, ST1, ST2 and ST3 were shown to be predominant in Guinea by representing more than 95% of the subtyped isolates. In parallel, the present study confirmed the overall scarcity of ST4 in Africa together with the low frequency of zoonotic transmission, represented herein by ST14 isolates likely inherited from bovid. Such a distribution together with the analysis of intra-ST diversity of the three main STs identified in Guinea strongly suggested a large-scale human-to-human transmission of ST3, while ST1 and ST2 were in large part probably also spread via various environmental sources in addition to the inter-human route. Finally, this large-scale study highlighted the importance of periodic screening and public health education for effective prevention of *Blastocystis* sp. especially regarding personal hygiene. Together with improved sanitation to reduce contact with contaminated water resources, all these measures may help the health authorities to prevent or reduce the prevalence and the risks of *Blastocystis* sp. infection in this highly endemic African region.

## Figures and Tables

**Table 1:** Distribution of participants according to their place of residence and the attendance to the two selected hospitals.

Residence Place	Confessional Health Center Anastasis	National Hospital Ignace Deen	Total
Boffa	0	1	1
Conakry	177	228	405
Coyah	0	2	2
Dubréka	0	12	12
Kamsar	0	2	2
Kassa	0	1	1
Kindia	72	2	74
Labé	0	1	1
Nzérékoré	1	0	1
Tanéré	0	1	1
**Total**	**250**	**250**	**500**

**Table 2:** Prevalence and ST distribution of *Blastocystis* sp. in the two Guinean hospitals screened in the present study.

Hospitals	Samples (n)	Positive Samples (n)	Prevalence(%)	*Blastocystis* sp. STs					
				ST1	ST2	ST3	ST4	ST14	MI ^a^
Confessional Health Center Anastasis	250	194	77.6%	62	27	49	1	7	48
National Hospital Ignace Deen	250	196	78.4%	34	39	68	1	3	51
**Total**	**500**	**390**	**78.0%**	**96**	**66**	**117**	**2**	**10**	**99**

^a^ MI, Mixed infections.

**Table 3:** Prevalence and ST distribution of *Blastocystis* sp. according to the residence place of participants.

Residence Place	Samples (n)	Positive Samples (n)	*Blastocystis* sp. STs					
			ST1	ST2	ST3	ST4	ST14	MI ^a^
Boffa	1	1	0	0	1	0	0	0
Conakry	405	314	69	54	100	1	10	80
Coyah	2	2	0	0	2	0	0	0
Dubréka	12	9	2	1	0	0	0	6
Kamsar	2	1	0	0	0	0	0	1
Kassa	1	1	0	0	1	0	0	0
Kindia	74	59	23	11	13	1	0	11
Labé	1	1	1	0	0	0	0	0
Nzérékoré	1	1	0	0	0	0	0	1
Tanéré	1	1	1	0	0	0	0	0
**Total**	**500**	**390**	**96**	**66**	**117**	**2**	**10**	**99**

^a^ MI, Mixed infections.

**Table 4:** Prevalence and ST distribution of *Blastocystis* sp. in African countries (completed from [10]).

African Region/Countries	Prevalence	Number of Subtyped Isolates	Subtyping Method	*Blastocystis* sp. STs									Mixed Infections ^d^	Reference
				ST1	ST2	ST3	ST4	ST5	ST6	ST7	ST10	ST14		
**North Africa**														
Tunisia	NA ^a^	61	Sequencing	18	10	31	1	0	0	1	0	0	0	[59]
Libya	28.0%	38	Sequencing	19	3	15	0	0	0	1	0	0	0	[39]
Libya	NA ^a^	48	Sequencing	26	13	9	0	0	0	0	0	0	0	[60]
Egypt	NA ^a^	36	PCR-STS ^b^	6	0	30	0	0	0	0	0	0	0	[61]
Egypt	NA ^a^	110	PCR-STS ^b^	15	0	49	0	0	33	13	0	0	0	[62]
Egypt	NA ^a^	33	Sequencing	0	0	33	0	0	0	0	0	0	0	[63]
Egypt	NA ^a^	21	Sequencing	4	4	13	0	0	0	0	0	0	0	[64]
Egypt	NA ^a^	44	PCR-STS ^b^	8	0	24	0	0	8	4	0	0	0	[65]
Egypt	NA ^a^	22	Sequencing	4	0	18	0	0	0	0	0	0	0	[66]
Egypt	NA ^a^	53	PCR-STS ^b^	16	4	30	3	0	0	0	0	0	0	[67]
Egypt	NA ^a^	100	RFLP ^c^	0	0	84	16	0	0	0	0	0	0	[68]
Egypt	NA ^a^	2	PCR-STS ^b^	1	0	1	0	0	0	0	0	0	0	[69]
Egypt	NA ^a^	51	PCR-STS	9	2	40	0	0	0	0	0	0	0	[70]
Egypt	NA ^a^	6	Sequencing	2	3	1	0	0	0	0	0	0	0	[71]
Algeria	NA ^a^	3	Sequencing	0	2	1	0	0	0	0	0	0	0	[72]
**Total**		**628**		**128**	**41**	**379**	**20**	**0**	**41**	**19**	**0**	**0**	**0**	
**West Africa**														
Nigeria	84.0%	127	Sequencing	51	42	33	0	0	0	1	0	0	0	[45]
Nigeria	49.0%	22	Sequencing	10	0	9	3	0	0	0	0	0	1	[39]
Nigeria	55.0%	18	Sequencing	8	4	5	0	0	1	0	0	0	0	[53]
Côte d’Ivoire	58.2%	64	Sequencing	32	14	18	0	0	0	0	0	0	0	[48]
Côte d’Ivoire	70.0%	0	NA ^a^	0	0	0	0	0	0	0	0	0	0	[51]
Côte d’Ivoire	87.0%	0	NA ^a^	0	0	0	0	0	0	0	0	0	0	[52]
Liberia	70.0%	25	Sequencing	7	7	8	3	0	0	0	0	0	5	[39]
Senegal	80.4%	453	Sequencing	113	226	107	0	0	0	3	2	2	135	[10]
Senegal	100%	103	Sequencing	29	21	51	2	0	0	0	0	0	0	[8]
Mali	49.7%	0	NA ^a^	0	0	0	0	0	0	0	0	0	0	[54]
**Guinea**	**78.0%**	**291**	**Sequencing**	**96**	**66**	**117**	**2**	**0**	**0**	**0**	**0**	**10**	**99**	**Present study**
**Total**		**1103**		**346**	**380**	**348**	**10**	**0**	**1**	**4**	**2**	**12**	**240**	
**Central Africa**														
Angola	25.6%	75	Sequencing	23	23	27	0	1	0	1	0	0	0	[55]
Cameroon	88.2%	65	Sequencing	23	9	33	0	0	0	0	0	0	0	[9]
Cameroon	75.4%	135	Metagenomics	45	32	57	1	0	0	0	0	0	0	[28]
**Total**		**275**		**91**	**64**	**117**	**1**	**1**	**0**	**1**	**0**	**0**	**0**	
**East Africa**														
Tanzania	81.8%	34	Metagenomics	11	13	10	0	0	0	0	0	0	0	[9]
Tanzania	55.6%	15	Metagenomics	1	12	2	0	0	0	0	0	0	0	[24]
Tanzania	60.9%	92	Sequencing	36	28	27	0	0	0	1	0	0	0	[50]
Tanzania	NA ^a^	6	Sequencing	1	3	2	0	0	0	0	0	0	0	[73]
Tanzania	53.0%	8	Sequencing	2	2	4	0	0	0	0	0	0	0	[53]
Sudan	47.0%	29	Sequencing	14	5	10	0	0	0	0	0	0	0	[53]
**Total**		**184**		**65**	**63**	**55**	**0**	**0**	**0**	**1**	**0**	**0**	**0**	
**Southern Africa**														
Madagascar	64.5%	158	Sequencing	80	36	42	0	0	0	0	0	0	13	[46]
Mozambique	14.1%	154	Sequencing	35	35	70	14	0	0	0	0	0	0	[56]
Malawi	69.6%	25	Metagenomics	7	6	12	0	0	0	0	0	0	0	[57]
**Total**		**337**		**122**	**77**	**124**	**14**	**0**	**0**	**0**	**0**	**0**	**13**	
**Grand total**		**2527**		**752**	**625**	**1023**	**45**	**1**	**42**	**25**	**2**	**12**	**253**	

^a^ NA, not applicable. ^b^ STS, subtype-specific sequence-tagged site. ^c^ RFLP, restriction fragment length polymorphism. ^d^ Mixed infections with undetermined STs.

## Data Availability

Not applicable.
